# Oily Fish Consumption and the Risk of Dyslipidemia in Korean Adults: A Prospective Cohort Study Based on the Health Examinees Gem (HEXA-G) Study

**DOI:** 10.3390/nu11102506

**Published:** 2019-10-17

**Authors:** Seong-Ah Kim, Jong-koo Lee, Daehee Kang, Sangah Shin

**Affiliations:** 1 Department of Food and Nutrition, Chung-Ang University, Gyeonggi-do 17546, Korea; sakim8864@gmail.com; 2 JW Lee Center for Global Medicine, Seoul National University College of Medicine, Seoul 03080, Korea; docmohw@snu.ac.kr; 3 Department of Family Medicine, Seoul National University Hospital, Seoul 03080, Korea; 4 Department of Preventive Medicine, Seoul National University College of Medicine, Seoul 03080, Korea; dhkang@snu.ac.kr; 5 Department of Biomedical Sciences, Seoul National University Graduate School, Seoul 03080, Korea; 6 Institute of Environmental Medicine, Seoul National University Medical Research Center, Seoul 03080, Korea

**Keywords:** fish, oily fish, omega-3 fatty acid, dyslipidemia, hypertriglyceridemia

## Abstract

Despite the beneficial effects of omega-3 fatty acids from fish or fish oil on cardiovascular diseases, limited information is available regarding the effects of oily fish in the diet on the risk of dyslipidemia. This study aimed to investigate the association between oily fish consumption and the incidence of dyslipidemia among Korean adults included in the Health Examinees Gem (HEXA-G) cohort during 5 years of follow-up. In total, 20,670 participants (5710 men and 14,960 women) were included in this study. The average intake of oily fish including dark meat fish, such as mackerel, pacific saury, and Spanish mackerel, and eel, was estimated using food frequency questionnaires. Oily fish consumption was associated with a significantly lower risk of hypertriglyceridemia in both men (Relative risk (RR) comparing extreme quintiles = 0.75; 95% CI 0.60–0.95; P for trend = 0.0121) and women (RR comparing extreme quintiles = 0.81; 95% CI 0.69–0.96; P for trend = 0.0110) after adjusting for potential confounders. In conclusion, increased consumption of oily fish was significantly associated with a lower risk of hypertriglyceridemia in the general Korean population. Future randomized clinical trials or prospective studies are required to confirm these findings in the Korean or other Asian populations.

## 1. Introduction

Dyslipidemia is a metabolic anomaly distinguished by an increase or reduction in the plasma lipid fraction [1]. Generally, high levels of low-density lipoprotein cholesterol (LDL-C), total cholesterol (TC), triglycerides (TG), and low levels of high-density lipoprotein cholesterol (HDL-C) are the primary risk factors of atherosclerosis, leading to cardiovascular disease (CVD) and coronary heart disease (CHD) [2]. The prevalence of dyslipidemia has continuously increased not only in Korea but also worldwide. An estimated 53% and 44% of the U.S. and Korean adults, respectively, have dyslipidemia [3,4]. Among the blood lipid components, LDL-C is the most important risk factor in the pathophysiology of CVD, and reducing LDL-C levels leads to dose-dependent reduction in the risk of major cardiovascular events that is proportional to the absolute magnitude of the reduction in LDL-C [5]. Furthermore, a meta-analysis of controlled trials reported that a 1 mmol/L reduction in TG was associated with reduction in coronary events by 54% for the overall population and by 43% in those with high TG levels [6]. Thus, improvement in the lipid profile is important to prevent CVD.

Nutritional and lifestyle modifications form the basis for treating dyslipidemia to prevent and reduce the risk of CVD [7]. Many epidemiological studies have reported the beneficial effects of omega-3 poly-unsaturated fatty acids (PUFAs) on cardiovascular health [8,9] The cardioprotective effects of omega-3 PUFAs, particularly in terms of blood lipid, can be explained by mechanisms such as increased clearance and decreased hepatic very-low-density lipoprotein production rates [8]. Fish is the primary dietary source of omega-3 PUFAs. Among omega-3 PUFAs, eicosapentaenoic acid (EPA), and docosahexaenoic acid (DHA) have been most widely studied for their beneficial effects on cardiovascular health [8,9]. Furthermore, these fatty acids reduce blood pressure, serum TG, and glucose levels, and increase HDL-C levels [10,11]. Other than PUFAs, such as DHA and EPA, fish also includes vitamin D and high-quality proteins, which may have beneficial effects against dyslipidemia [12,13].

Despite numerous epidemiological studies on the beneficial effects of omega-3 fatty acids from fish or fish oil against CVD, limited information is available regarding the effects of oily fish in the diet on the risk of dyslipidemia. Oily fish, such as mackerel, tuna, salmon, sardines, and herring, are the primary dietary sources of omega-3 fatty acids, particularly DHA and EPA [8,9]. Therefore, oily fish may have more potent effects on the risk of dyslipidemia when compared to non-oily fish.

Moreover, although fish is a major part of the Korean diet, accounting for approximately 20% of the energy intake from animal sources owing to its peninsular characteristics [14], limited information is available regarding the effects of fish consumption against the risk of dyslipidemia. The purpose of the present study was to examine the association between oily fish consumption and the incidence of dyslipidemia among Korean adults included in the Health Examinees Gem (HEXA-G) Study cohort during 5 years of follow-up.

## 2. Materials and Methods

### 2.1. Study Population

The HEXA study [15,16] is a large-scale community-based prospective cohort study conducted in Korea. The baseline survey of HEXA study was conducted from 2004 to 2013. In total, 169,722 participants aged 40–69 years were recruited in 38 general hospitals and health examination centers in eight regions in Korea. Based on previous HEXA studies, this study used data of the individuals included in the HEXA-G cohort, for which additional eligibility criteria were applied (i.e., hospitals or health examination centers). Of the original 38 sites, 30,374 individuals at 21 sites were excluded in accordance with previously reported exclusion criteria [17]. Thus far, 64,486 participants of the HEXA-G study completed the initial follow-up survey between 2012 and 2016. Among them, those with dyslipidemia (*n =* 40,983), diabetes (*n =* 1,277), or cardiovascular diseases (*n =* 597) at baseline and with insufficient data regarding blood lipid profiles at baseline (*n =* 3) and at follow-up (*n =* 6), implausible energy intake (< 800 or ≥ 4000 kcal/day in men and < 500 or ≥ 3500 kcal/day in women) (*n =* 938), and those with insufficient information regarding body mass index (BMI) (*n =* 12) were excluded. Finally, 20,670 participants (5710 men and 14,960 women) were included in this study. The HEXA-G study was approved by the Ethics Committee of the Korean Health and the institutional review boards of all participating hospitals (IRB No. E-1503-103-657). All participants provided informed written consent prior to participating the study.

### 2.2. Diet Assessment

The validated semi-quantitative food frequency questionnaire (FFQ), including 106 food items, was used at baseline and follow-up examinations to assess oily fish consumption and nutrient intake among participants. The participants were asked how often they consumed each food item on average during the past year. Daily nutrient intake was calculated by multiplying the frequency of consumption of each food item by its nutrient content and summing up the nutrient intake from all food items. Of the 14 fish items, oily fish comprised dark-meat fish, including mackerel, pacific saury, and Spanish mackerel, and eel. The cumulative average intake of oily fish was estimated using the first (at baseline) and second (at follow-up) FFQs to represent long-term intake.

### 2.3. Definition of Dyslipidemia

The end point for this study was the occurrence of dyslipidemia and its components, including hypercholesterolemia, hyper-LDL cholesterolemia, hypo-HDL cholesterolemia, and hypertriglyceridemia occurring after baseline but before follow-up examination. Dyslipidemia and its components were diagnosed on the basis of the analysis of blood samples obtained after 8 h of fasting.

Participants satisfying one of the following four components were diagnosed as having dyslipidemia. Hypercholesterolemia was defined as a blood TC level ≥ 200 mg/dL; hyper-LDL cholesterolemia as blood LDL-C ≥ 130 mg/dL; hypo-HDL cholesterolemia as HDL-C level < 40 mg/dL; and hypertriglyceridemia as TG level ≥ 150 mg/dL.

### 2.4. Assessment of Other Variables

Body mass index (BMI) was determined by dividing the body weight by the square of the height (kg/m^2^). BMI values were used to classify participants into the following groups: underweight (BMI < 18.5 kg/m^2^), normal (BMI ≥ 18.5 kg/m^2^ and < 23 kg/m^2^), overweight (BMI ≥ 23 kg/m^2^ and < 25 kg/m^2^), and obese (BMI ≥ 25 kg/m^2^) in accordance with the International Obesity Task Force for adults in Asian and Pacific regions [18]. Sociodemographic variables, such as age, sex, and education level, and lifestyle variables, such as alcohol consumption, current smoking status, and physical activity, were obtained through self-administered questionnaires. Education level was divided into three categories: under middle school, high school, and over college. Alcohol consumption was divided into two categories: “current drinker” (drank alcohol at the time of survey) or “non-drinker” (never drank alcohol or have abstained from alcohol). Current smoking status was divided into three categories: “current smoker” (smoked cigarettes at the time of survey), “past smoker” (have abstained from cigarettes smoking), or “never smoker” (never smoked cigarettes). Physical activity was divided into two categories: “active” (performed physical activity for ≥ 30 min once a day for ≥ 5 d a week) or “inactive.”

### 2.5. Statistical Analysis

For each participant, person-years of follow-up were calculated from baseline to the first follow-up examination. We calculated the incidence of dyslipidemia and its components per person years. We performed Cox proportional hazards regression analysis to estimate the relative risks (RRs) and 95% confidence interval (CI) of dyslipidemia in accordance with the quintiles of oily fish consumption after adjusting for potential confounders, such as age (continuous), BMI (continuous), education level, alcohol consumption, current smoking status, physical activity, and energy intake (continuous) by sex. Subgroup analysis was performed for dyslipidemia and its components, which revealed significant associations with oily fish consumption. We estimated the RR and 95% CI of the fifth quintile (Q5) in comparison with the first quintile (Q1) by sex in accordance with baseline age, baseline BMI level, and menopausal status (in women). All statistical analyses were performed using SAS software version 9.4 (SAS Institute, Cary, NC, USA) and a two-sided p-value < 0.05 was considered statistically significant.

## 3. Results

During the 5 years of follow-up (total 104,927 person-years), we documented 8751 incident cases of dyslipidemia (7127 cases of hypercholesterolemia, 3923 cases of hyper-LDL cholesterolemia, 674 cases of hypo-HDL cholesterolemia, and 2287 cases of hypertriglyceridemia).

The general characteristics of the study population at baseline in accordance with the quintiles of oily fish consumption by sex are shown in Table 1. In both men and women, participants with higher oily fish consumption tended to have a higher education level and higher BMI level, consume alcohol, performe physical activity, and consume more energy and macronutrients, including carbohydrate, protein, and fat, in comparison with those with a lower consumption (all P < 0.001).

Table 2 shows the RRs of dyslipidemia in accordance with the quintiles of oily fish consumption by sex. The RR for hypertriglyceridemia among individuals with the highest oily fish consumption was 0.75 (95% CI 0.60–0.95, P for trend = 0.0121) in men and 0.81 (95% CI 0.69–0.96, P for trend = 0.0110) in women after adjusting for age, BMI, education level, alcohol consumption, current smoking status, physical activity, and energy intake in comparison with those for individuals with the lowest consumption. However, there was no significant association between oily fish consumption and other components of dyslipidemia.

Table 3 shows the RRs of dyslipidemia in accordance with the quintiles of total fish consumption by sex. Although a significant positive trend was observed between total fish consumption and the risk of hyper-LDL cholesterolemia in men after adjusting for potential confounders (P for trend = 0.0074), no association was observed between total fish consumption and the risk of dyslipidemia and its components.

**Figure 1:**
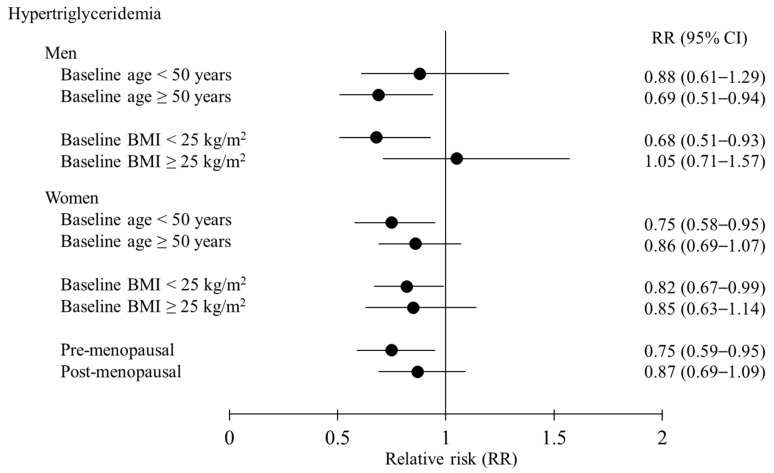
Relative risks of hypertriglyceridemia when comparing extreme quintiles of oily fish consumption by sex in accordance with baseline age, baseline BMI level, and menopausal status (in women) in the HEXA-G study.

Figure 1 shows the RRs of hypertriglyceridemia on comparing extreme quintiles of oily fish consumption by sex in accordance with baseline age, baseline BMI level, and menopausal status (in women). The group with the highest oily fish consumption with a baseline age ≥ 50 years among men displayed a 31% lower risk of hypertriglyceridemia in comparison with the group with the lowest oily fish consumption, whereas, among women, the group with the highest oily fish consumption with a baseline age < 50 years displayed a 25% lower risk of hypertriglyceridemia in comparison with the group with the lowest oily fish consumption. Among both men and women, the group with the highest oily fish consumption with a baseline BMI < 25 kg/m^2^ displayed a 32% and 18%, respectively, lower risk of hypertriglyceridemia in comparison with the lowest oily fish consumption group. Among pre-menopausal women, the RR for hypertriglyceridemia in the group with the highest oily fish consumption was 25% lower than that of the group with the lowest consumption.

## 4. Discussion

This prospective cohort study, conducted on a population sample from Korea, a country with high fish consumption, shows a significant inverse association between oily fish consumption and the incidence of hypertriglyceridemia after a 5-year follow-up.

Numerous randomized controlled trials (RCTs) have reported that either a fish in the diet or fish oil supplements improve blood TG levels in healthy individuals or those with hyperlipidemia [8,9] and those with type 2 diabetes [19,20]. The present results showed no association between total fish consumption and the risk of dyslipidemia; however, a significant inverse association was observed between oily fish consumption and the risk of hypertriglyceridemia. The TG-lowering effect of fish consumption depends on the long chain omega-3 PUFA content in fish [8]. Therefore, the intake of oily fish high in omega-3 fatty acids, rather than total fish, can reduce risk of hypertriglyceridemia. The potential mechanism underlying the hypotriglyceridemic effect of oily fish can be explained on the basis of the role of omega-3 fatty acids in the inhibition of TG and hepatic very-low-density lipoprotein synthesis [21,22]. Because high blood TG levels are independent risk factors for CVD, such as CHD, ischemic stroke, and myocardial infarction [6], oily fish consumption can be beneficial for cardiovascular health.

Subgroup analyses (Figure 1) revealed that the TG-lowering effects of oily fish consumption were observed only at a baseline BMI < 25 kg/m^2^ (non-obese) and in pre-menopausal women. Elevated TG level is the most prominent feature of dyslipidemia in obesity [23]. The overload of hepatic TG leads to delayed clearance of the TG-rich lipoproteins and the formation of small dense LDL aggregates, thus increasing TG levels and subsequently resulting in dyslipidemia [23]. Thus, impaired lipid metabolism in obesity may negatively affect the TG-lowering effects of oily fish, resulting in a null association in obese individuals. Furthermore, postmenopausal women tend to have significantly different lipid profiles, higher concentrations of TC, TG, and LDL-C, but lower HDL-C levels in comparison with premenopausal women [24]. Owing to these differences, these associations may be significant only among pre-menopausal women.

However, although omega-3 PUFAs usually decrease blood TG levels, their effects on TC, LDL-C, and HDL-C levels are subtle and conflicting [25]. A systematic review and meta-analysis that summarized 47 RCTs reported that fish oil supplementation significantly reduced blood TG levels but not TC, HDL-C, or LDL-C levels in hyperlipidemia patients [26]. Rather, LDL-C levels increased slightly but significantly after fish oil supplementation [26]. Similarly, in the present study, oily fish consumption did not significantly alter the risk of hypercholesterolemia, hyper-LDL cholesterolemia, and hypo-HDL cholesterolemia; however, a significant positive trend was observed between total fish consumption and the risk of hyper-LDL cholesterolemia.

Although the beneficial effects of omega-3 fatty acids from fish or fish oil against CVD risk factors, including dyslipidemia, have been widely reported, limited information is available regarding the effects of oily fish consumption on the risk of dyslipidemia. In an 8-week RCT in China, oily fish consumption, including salmon, significantly reduced serum TG levels and increased HDL-C levels in adult men with hypertriglyceridemia [27], while it did not alter blood lipid levels among Chinese adult women with hypertriglyceridemia in the subsequent 8-week trial [28]. However, in the present study, a higher consumption of oily fish, including dark-meat fish (mackerel, pacific saury, and Spanish mackerel) and eel, was associated with a lower risk of hypertriglyceridemia; however, it was not associated with the risk of hypercholesterolemia, hyper-LDL cholesterolemia, and hypo-HDL cholesterolemia. The differences in oily fish species, content of EPA + DHA in fish, and study population among these studies may have resulted in these inconsistencies.

This may lead to questions regarding the optimal source of omega-3 fatty acids for foods or supplements. The optimal dose of omega-3 PUFAs to reduce the risk of dyslipidemia is yet unclear. Fish, especially oily fish, contains not only sufficient omega-3 PUFAs but also various nutrients, including high-quality proteins, vitamin D, selenium, and other minerals and elements [9]. Some nutrients in fish, such as vitamin E and selenium, also have beneficial roles to offset the toxic effects of methylmercury, an environmental contaminant found in fish [29,30]. Therefore, the consumption of fish, especially oily fish, on a regular basis is recommended for cardiovascular health [8,31]. Moreover, according to the latest scientific evidence, omega-3 fatty acid supplements are not useful, thus are not recommended for the prevention of CVD [32,33]. However, some large predatory fish, such as shark, sword-fish, tilefish, king mackerel, and bigeye tuna, have high levels of methylmercury, which may have neurotoxic effects in the fetus and reduce cognition in young children; hence, it is recommended to consume an optimal amount (1–2 servings a week) of fish so that the benefits can outweigh the risks, particularly for pregnant women and children [8].

The results of this study disclose that the blood lipid benefits were strongest for oily fish, such as mackerel, pacific saury, and eel, compared to leaner fish, including yellow croaker, flat fish, and hair tail. Though all kinds of fish are highly nutritious due to high contents of protein, fat soluble vitamins, and minerals, for preventing dyslipidemia and promoting cardiovascular health, regular consumption of oily fish is recommended.

This study had several limitations. First, we could not include all types of oily fish owing to predetermined lists of food items in the FFQ. Second, owing to the lack of an omega-3 fatty acids database, the intake of omega-3 fatty acids from fish could not be considered a covariable. Third, the effects of methylmercury on fish cannot be excluded, since methylmercury may attenuate the health benefits of omega-3 fatty acids [34]. However, the current evidence suggests that the benefits of consuming 1 to 2 servings of fish per week outweigh the risks, especially if a variety of seafood are consumed [8]. Further studies are required to examine the comprehensive effects of omega-3 fatty acids and methylmercury in fish on the risk of dyslipidemia.

Despite these limitations, this is the first prospective study to examine the association between oily fish consumption and the risk of dyslipidemia in the Korean population. Because of the prospective study design, we examined the causal association between oily fish consumption and the risk of dyslipidemia. The strengths of the present study using the HEXA-G sample include the relative homogeneity of the cohort, allowing for increased internal validity. Furthermore, we used a validated FFQ, which ensured standardized protocols to obtain information regarding the participants’ long-term dietary intake.

## 5. Conclusions

Increased oily fish consumption is significantly associated with a lower risk of hypertriglyceridemia after adjusting for potential confounders in the general Korean population. The present results suggest that oily fish consumption potentially protects against dyslipidemia and related chronic diseases among Korean adults. Future randomized clinical trials or prospective studies are needed to further analyze these findings in the Korean or other Asian populations.

## Figures and Tables

**Table 1:** General characteristics of the study population at baseline examination in accordance with the quintiles of oily fish consumption by sex in the HEXA-G study.

	Men						Women					
	Q1	Q2	Q3	Q4	Q5	*p* Value	Q1	Q2	Q3	Q4	Q5	*p* value
N	1120	1146	1239	1089	1116		3044	2940	2989	2983	3004	
Age (years)	55.2 ± 8.6	54.2 ± 8.2	54.7 ± 8.2	53.9 ± 8.5	53.8 ± 8.1	<0.0001	50.2 ± 7.5	49.7 ± 7.2	50.0 ± 7.4	49.4 ± 7.1	50.3 ± 7.0	<0.0001
Age (years), *n* (%)												
40–49	312 (27.9)	348 (30.4)	351 (28.3)	361 (33.2)	365 (32.7)	<0.0010	1564 (51.4)	1561 (53.1)	1536 (51.4)	1654 (55.5)	1491 (49.6)	<0.0001
50–59	389 (34.7)	435 (38.0)	477 (38.5)	405 (37.2)	423 (37.9)		1044 (34.3)	1045 (35.5)	1065 (35.6)	1005 (33.7)	1145 (38.1)	
60–69	419 (37.4)	363 (31.7)	411 (33.2)	323 (29.7)	328 (29.4)		436 (14.3)	334 (11.4)	388 (13.0)	324 (10.9)	368 (12.3)	
Education level, *n* (%)												
Under middle school	321 (28.7)	241 (21.0)	272 (22.0)	213 (19.6)	184 (16.5)	<0.0001	1032 (33.9)	811 (27.6)	874 (29.2)	738 (24.7)	751 (25.0)	<0.0001
High school	413 (36.9)	473 (41.3)	485 (39.1)	431 (39.6)	472 (42.3)		1314 (43.2)	1382 (47.0)	1374 (46.0)	1493 (50.1)	1497 (49.8)	
Over college	370 (33.0)	419 (36.6)	466 (37.6)	435 (39.9)	448 (40.1)		665 (21.9)	716 (24.4)	706 (23.6)	729 (24.4)	719 (23.9)	
BMI (kg/m^2^)	23.2 ± 2.7	23.4 ± 2.6	23.5 ± 2.6	23.8 ± 2.6	23.9 ± 2.6	<0.0001	22.7 ± 2.7	22.9 ± 2.8	22.9 ± 2.7	23.0 ± 2.7	23.1 ± 2.7	<0.0001
Obesity, *n* (%)												
Underweight	37 (3.3)	29 (2.5)	26 (2.1)	15 (1.4)	20 (1.8)	<0.0001	126 (4.1)	94 (3.2)	81 (2.7)	78 (2.6)	66 (2.2)	0.0003
Normal	497 (44.4)	476 (41.5)	500 (40.4)	425 (39.0)	407 (36.5)		1623 (53.3)	1590 (54.1)	1577 (52.8)	1571 (52.7)	1525 (50.8)	
Overweight	314 (28.0)	350 (30.5)	393 (31.7)	301 (27.6)	301 (27.0)		706 (23.2)	680 (23.1)	743 (24.9)	749 (25.1)	779 (25.9)	
Obese	272 (24.3)	291 (25.4)	320 (25.8)	348 (32.0)	388 (34.8)		589 (19.4)	576 (19.6)	588 (19.7)	585 (19.6)	634 (21.1)	
Alcohol consumption												
Non-drinker	377 (33.7)	298 (26.0)	332 (26.8)	252 (23.1)	254 (22.8)	<0.0001	2104 (69.1)	1925 (65.5)	1966 (65.8)	1893 (63.5)	2008 (66.8)	0.0008
Current drinker	740 (66.1)	847 (73.9)	901 (72.7)	831 (76.3)	857 (76.8)		929 (30.5)	1001 (34.1)	1011 (33.8)	1081 (36.2)	979 (32.6)	
Current smoking status												
Never smoker	410 (36.6)	423 (36.9)	436 (35.2)	364 (33.4)	366 (32.8)	0.5620	2955 (97.1)	2858 (97.2)	2914 (97.5)	2910 (97.6)	2908 (96.8)	0.5502
Past smoker	448 (40.0)	458 (40.0)	508 (41.0)	445 (40.9)	455 (40.8)		28 (0.9)	29 (1.0)	23 (0.8)	24 (0.8)	37 (1.2)	
Current smoker	258 (23.0)	261 (22.8)	289 (23.3)	276 (25.3)	287 (25.7)		51 (1.7)	40 (1.4)	40 (1.3)	39 (1.3)	40 (1.3)	
Physical activity												
Active	217 (19.4)	224 (19.6)	241 (19.5)	237 (21.8)	288 (25.8)	0.0002	485 (15.9)	467 (15.9)	522 (17.5)	520 (17.4)	651 (21.7)	<0.0001
Inactive	885 (79.0)	895 (78.1)	960 (77.5)	819 (75.2)	793 (71.1)		2519 (82.8)	2426 (82.5)	2425 (81.1)	2390 (80.1)	2274 (75.7)	
Nutrient intake												
Total energy (Kcal/d)	1658.6 ± 436.3	1754.3 ± 437.2	1816.8 ± 406.4	1913.0 ± 460.6	2057.5 ± 492.8	<0.0001	1534.4 ± 431.2	1600.8 ± 445.5	1678.4 ± 456.7	1758.0 ± 481.0	1921.0 ± 7.0	<0.0001
Carbohydrates (g/d)	309.0 ± 76.8	317.6 ± 77.6	326.5 ± 72.4	335.4 ± 78.4	348.8 ± 80.1	<0.0001	286.4 ± 79.8	292.9 ± 82.3	301.4 ± 82.1	310.2 ± 85.5	327.3 ± 90.6	<0.0001
Protein (g/d)	49.3 ± 16.8	56.0 ± 17.6	59.5 ± 16.9	65.6 ± 20.5	76.7 ± 26.0	<0.0001	46.7 ± 16.0	51.1 ± 16.6	56.0 ± 18.5	60.8 ± 19.4	72.2 ± 25.3	<0.0001
Fat (g/d)	22.3 ± 13.1	26.3 ± 13.0	27.8 ± 12.4	32.0 ± 14.8	37.4 ± 17.1	<0.0001	20.6 ± 11.6	23.2 ± 12.1	26.0 ± 13.3	29.0 ± 140	34.8 ± 17.0	< .0001

HEXA-G: Health Examinees Gem.

**Table 2:** Relative risks of dyslipidemia in accordance with the quintiles of oily fish consumption by sex in the HEXA-G study.

	Oily Fish Consumption (g/d)					
	Q1	Q2	Q3	Q4	Q5	P for Trend
**Men (*n =* 5710)**						
Median (range)	1.7 (0.0–2.6)	3.5 (2.7–4.9)	6.0 (5.0-6.8)	8.6 (6.8-10.7)	16.0 (10.7-116.4)	
Person-years, mean (sum)	5.0 (5579.4)	4.9 (5658.3)	5.0 (6138.7)	4.9 (5350.0)	5.0 (5589.6)	
Hypercholesterolemia						
Cases, *n*	287	292	313	275	313	
Model 1	1.00 (Ref.)	1.00 (0.85-1.18)	0.97 (0.83-1.14)	1.00 (0.85-1.19)	1.06 (0.90-1.24)	0.3829
Model 2	1.00 (Ref.)	0.99 (0.84-1.17)	0.96 (0.82-1.13)	0.99 (0.84-1.17)	1.04 (0.88-1.23)	0.5078
Hyper-LDL cholesterolemia						
Cases, *n*	164	155	168	174	186	
Model 1	1.00 (Ref.)	0.92 (0.74-1.15)	0.90 (0.73-1.12)	1.11 (0.89-1.37)	1.09 (0.88-1.35)	0.1160
Model 2	1.00 (Ref.)	0.92 (0.74-1.15)	0.90 (0.72-1.12)	1.11 (0.89-1.38)	1.10 (0.88-1.37)	0.1072
Hypo-HDL cholesterolemia						
Cases, *n*	65	51	80	55	47	
Model 1	1.00 (Ref.)	0.75 (0.52-1.09)	1.06 (0.76-1.47)	0.84 (0.59-1.21)	0.66 (0.45-0.96)	0.0507
Model 2	1.00 (Ref.)	0.79 (0.55-1.14)	1.11 (0.79-1.54)	0.87 (0.60-1.27)	0.68 (0.46-1.01)	0.0762
Hypertriglyceridemia						
Cases, *n*	159	166	175	163	146	
Model 1	1.00 (Ref.)	0.99 (0.80-1.23)	0.93 (0.75-1.16)	0.99 (0.79-1.23)	0.80 (0.64-1.01)	0.0419
Model 2	1.00 (Ref.)	0.98 (0.78-1.21)	0.91 (0.73-1.13)	0.94 (0.75-1.17)	0.75 (0.60-0.95)	0.0121
Dyslipidemia						
Cases, *n*	418	420	462	411	431	
Model 1	1.00 (Ref.)	0.98 (0.86-1.12)	0.98 (0.85-1.11)	1.01 (0.89-1.16)	0.98 (0.86-1.13)	0.9710
Model 2	1.00 (Ref.)	0.97 (0.85-1.12)	0.97 (0.84-1.11)	1.00 (0.87-1.14)	0.96 (0.84-1.11)	0.7353
**Women (*n =* 14,960)**						
Median (range)	1.5 (0.0-2.3)	3.3 (2.3-4.3)	5.8 (4.3-6.7)	8.5 (6.8-10.7)	15.0 (10.7-102.1)	
Person-years, mean (sum)	5.0 (15232.7)	5.1 (14975.6)	5.1 (15171.8)	5.1 (15261.0)	5.3 (15969.5)	
Hypercholesterolemia						
Cases, *n*	1104	1080	1081	1156	1226	
Model 1	1.00 (Ref.)	0.97 (0.89-1.06)	0.95 (0.87-1.03)	1.02 (0.94-1.11)	0.97 (0.89-1.05)	0.7576
Model 2	1.00 (Ref.)	0.96 (0.89-1.05)	0.94 (0.86-1.02)	1.00 (0.92-1.09)	0.97 (0.89-1.05)	0.8116
Hyper-LDL cholesterolemia						
Cases, *n*	603	598	575	617	683	
Model 1	1.00 (Ref.)	0.98 (0.88-1.10)	0.91 (0.81-1.02)	0.99 (0.88-1.11)	0.97 (1.01-1.02)	0.7691
Model 2	1.00 (Ref.)	0.97 (0.87-1.09)	0.91 (0.81-1.02)	0.98 (0.88-1.10)	0.97 (0.87-1.09)	0.9111
Hypo-HDL cholesterolemia						
Cases, *n*	76	81	70	72	77	
Model 1	1.00 (Ref.)	1.05 (0.77-1.44)	0.87 (0.63-1.20)	0.91 (0.66-1.26)	0.84 (0.61-1.16)	0.1915
Model 2	1.00 (Ref.)	1.05 (0.77-1.44)	0.87 (0.63-1.20)	0.91 (0.66-1.26)	0.83 (0.60-1.15)	0.1699
Hypertriglyceridemia						
Cases, *n*	320	297	294	267	300	
Model 1	1.00 (Ref.)	0.92 (0.78-1.08)	0.87 (0.74-1.02)	0.80 (0.68-0.95)	0.79 (0.67-0.92)	0.0024
Model 2	1.00 (Ref.)	0.92 (0.78-1.08)	0.88 (0.75-1.04)	0.81 (0.69-0.96)	0.81 (0.69-0.96)	0.0110
Dyslipidemia						
Cases, *n*	1299	1294	1263	1329	1424	
Model 1	1.00 (Ref.)	0.99 (0.92-1.07)	0.94 (0.87-1.01)	0.99 (0.92-1.07)	0.95 (0.88-1.02)	0.2516
Model 2	1.00 (Ref.)	0.98 (0.91-1.06)	0.93 (0.86-1.01)	0.98 (0.91-1.06)	0.95 (0.88-1.03)	0.3061

HEXA-G: Health Examinees Gem; RR (95% CI); Model 1: Adjusted for age (continuous), and BMI (continuous); Model 2: Additionally adjusted for education level (under middle school, high school, or over college), smoking status (never, past, or current smoker), alcohol consumption (non-drinker or current drinker), physical activity (yes or no), energy intake (continuous).

**Table 3:** Relative risks of dyslipidemia in accordance with the quintiles of total fish consumption by sex in the HEXA-G study.

	Total Fish Consumption (g/d)					
	Q1	Q2	Q3	Q4	Q5	P for Trend
**Men (*n =* 5710)**						
Median (range)	11.9 (0.2-16.8)	21.0 (16.8–25.1)	29.6 (25.1–34.8)	40.8 (34.8–49.9)	65.5 (49.9–292.2)	
Person-years, mean (sum)	5.0 (5468.7)	4.9 (5472.3)	4.9 (5553.5)	5.0 (5870.7)	4.9 (5950.8)	
Hypercholesterolemia						
Cases, *n*	261	279	308	319	313	
Model 1	1.00 (Ref.)	1.05 (0.88–1.24)	1.14 (0.97–1.35)	1.13 (0.96–1.33)	1.12 (0.95–1.32)	0.1948
Model 2	1.00 (Ref.)	1.04 (0.88–1.23)	1.13 (0.96–1.34)	1.12 (0.95–1.32)	1.11 (0.93–1.33)	0.2593
Hyper-LDL cholesterolemia						
Cases, *n*	151	140	169	192	195	
Model 1	1.00 (Ref.)	0.91 (0.72–1.15)	1.08 (0.87–1.35)	1.17 (0.95–1.45)	1.20 (0.97–1.49)	0.0145
Model 2	1.00 (Ref.)	0.91 (0.72–1.15)	1.10 (0.88–1.37)	1.19 (0.96–1.49)	1.25 (1.00–1.58)	0.0074
Hypo-HDL cholesterolemia						
Cases, *n*	68	53	48	74	55	
Model 1	1.00 (Ref.)	0.77 (0.53–1.10)	0.68 (0.47–0.99)	0.99 (0.71–1.37)	0.72 (0.50–1.03)	0.3026
Model 2	1.00 (Ref.)	0.80 (0.56–1.15)	0.74 (0.51–1.08)	1.03 (0.73–1.44)	0.72 (0.49–1.06)	0.3069
Hypertriglyceridemia						
Cases, *n*	157	148	147	188	169	
Model 1	1.00 (Ref.)	0.90 (0.71–1.12)	0.89 (0.71–1.11)	1.06 (0.86–1.31)	0.93 (0.75–1.15)	0.9930
Model 2	1.00 (Ref.)	0.89 (0.71–1.12)	0.88 (0.70–1.11)	1.02 (0.82–1.27)	0.87 (0.69–1.10)	0.5172
Dyslipidemia						
Cases, *n*	396	403	409	478	456	
Model 1	1.00 (Ref.)	1.00 (0.87–1.15)	1.00 (0.87–1.15)	1.11 (0.97–1.27)	1.06 (0.92–1.21)	0.2005
Model 2	1.00 (Ref.)	0.99 (0.86-1.14)	0.99 (0.86–1.14)	1.09 (0.95–1.26)	1.04 (0.90–1.20)	0.3559
**Women (*n =* 14,960)**						
Median (range)	11.9 (0.0-16.8)	21.0 (16.8–25.1)	29.6 (25.1–34.8)	41.0 (34.8–49.9)	65.5 (49.9–348.5)	
Person-years, mean (sum)	5.1 (15389.3)	5.1 (15350.3)	5.1 (15201.3)	5.1 (15218.5)	5.3 (15451.2)	
Hypercholesterolemia						
Cases, *n*	1099	1082	1152	1158	1156	
Model 1	1.00 (Ref.)	0.98 (0.90–1.06)	1.08 (0.99–1.17)	1.04 (0.96–1.13)	0.99 (0.91–1.07)	0.9380
Model 2	1.00 (Ref.)	0.97 (0.90–1.06)	1.07 (0.99–1.17)	1.04 (0.95-1.13)	1.00 (0.91–1.09)	0.9228
Hyper-LDL cholesterolemia						
Cases, *n*	586	582	631	631	646	
Model 1	1.00 (Ref.)	0.98 (0.88–1.10)	1.11 (0.99–1.24)	1.06 (0.95–1.19)	1.02 (0.91–1.14)	0.6460
Model 2	1.00 (Ref.)	0.99 (0.88–1.11)	1.11 (0.99–1.25)	1.07 (0.95–1.20)	1.05 (0.93–1.18)	0.4159
Hypo-HDL cholesterolemia						
Cases, *n*	87	71	64	73	81	
Model 1	1.00 (Ref.)	0.82 (0.60–1.12)	0.76 (0.55–1.05)	0.83 (0.60–1.13)	0.84 (0.62–1.14)	0.5000
Model 2	1.00 (Ref.)	0.81 (0.59–1.10)	0.76 (0.55–1.05)	0.81 (0.59–1.12)	0.82 (0.59–1.14)	0.4593
Hypertriglyceridemia						
Cases, *n*	307	304	297	273	297	
Model 1	1.00 (Ref.)	0.99 (0.84–1.16)	1.00 (0.85–1.17)	0.88 (0.75–1.03)	0.88 (0.75–1.04)	0.0537
Model 2	1.00 (Ref.)	1.00 (0.85–1.17)	1.02 (0.87–1.20)	0.90 (0.76–1.07)	0.93 (0.78–1.10)	0.2375
Dyslipidemia						
Cases, *n*	1299	1282	1344	1338	1346	
Model 1	1.00 (Ref.)	0.98 (0.91–1.06)	1.06 (0.98–1.15)	1.02 (0.94–1.10)	0.97 (0.90–1.05)	0.4501
Model 2	1.00 (Ref.)	0.98 (0.91–1.06)	1.06 (0.98–1.15)	1.02 (0.94–1.10)	0.98 (0.90–1.06)	0.6362

HEXA-G: Health Examinees Gem; RR (95% CI); Model 1: Adjusted for age (continuous), and BMI (continuous); Model 2: Additionally adjusted for education level (under middle school, high school, or over college), smoking status (never, past, or current smoker), alcohol consumption (non-drinker or current drinker), physical activity (yes or no), energy intake (continuous).
